# Supplementary material for: The neural representation of personally familiar and unfamiliar faces in the distributed system for face perception
Source: Sci Rep. 2017 Sep 25;7:12237. doi: 10.1038/s41598-017-12559-1 (PMC5612994; doi:10.1038/s41598-017-12559-1)
Supplement: Supplementary file 1 — Supplementary Material [file 41598_2017_12559_MOESM1_ESM.pdf]

Supplementary Material

The neural representation of personally familiar and unfamiliar faces in the distributed system for face perception

Matteo Visconti di Oleggio Castello, Yaroslav O. Halchenko, J. Swaroop Guntupalli, Jason D. Gors, M. Ida Gobbini

Supplementary Methods 1
- Movie data fMRI acquisition and preprocessing 1
- Permutations for Identity and Familiarity decoding 2
- Early visual cortex ROI analysis and HMAX features 3
- Quantification of similarities within and between core and extended systems 4
- Comparison of second-order representational geometries obtained from task data and movie data 4
- Model-based RSA within systems 5
- Distances of EV ROIs from dorsal and ventral systems 5

Supplementary Tables 6

Supplementary Figures 10

Supplementary References 26

Supplementary Methods

Movie data fMRI acquisition and preprocessing

Eleven subjects watched the full-length feature film "Raiders of the Lost Ark" divided into eight parts of ~14 min 20 s duration. Successive parts repeated the final 20s of the previous part to account for hemodynamic response. Data collected during overlapping movie segments were discarded from the beginning of each part. Participants viewed the first four parts of the movie in one session and were taken out of the scanner for a short break. Participants then viewed the remaining four parts after the break. Video was projected onto a rear projection screen with an LCD projector, which the subject viewed through a mirror on the head coil. The video image subtended a visual angle of ~22.7° horizontally and 17° vertically. Audio was presented through MR-compatible headphones. Subjects were instructed to pay attention to the movie and enjoy (Guntupalli et al., 2016; Haxby et al., 2011). Additional details on the MR parameters can be found in the Supplementary material.

Subjects were scanned in a Philips Intera Achieva 3T scanner with an 8 channel head coil at the Dartmouth Brain Imaging Center (original and preprocessed data could be

obtained from http://datasets.datalad.org/?dir=/labs/haxby/raiders). Functional scans were acquired with an echo planar imaging sequence (TR=2.5 s, TE=35 ms, flip angle = 90°, 80 x 80 matrix, FOV=240 mm x 240 mm) every 2.5 s with whole brain coverage (41x3 mm thick interleaved axial slices). We acquired a total of 2718 functional scans with 1350 TRs in four runs during the first session and 1368 TRs in four runs during the second session. T1-weighted anatomical scans were acquired at the end of each session (MPRAGE, TR=9.85 s, TE=4.53 s, flip angle=8°, 256 x 256 matrix, FOV=240 mm, 160 1 mm thick sagittal slices). The voxel resolution was 0.938 mm x 0.938 mm x 1.0 mm. Each subject's fMRI data were preprocessed using AFNI software (Cox, 1996; http://afni.nimh.nih.gov). Functional data were corrected for the order of slice acquisition then for head motion by aligning to the last volume of the last functional run. Any spikes in the data were removed using 3dDespike in AFNI. Data were then filtered using 3dBandpass in AFNI to remove any temporal signal variation slower than 0.00667 Hz, faster than 0.1 Hz, and that correlated with the whole brain average signal or the head movement parameters. Residual data were then aligned to the MNI 152 brain template using nearest neighbor resampling and spatially smoothed with a 4 mm full-width-at-half-maximum (FWHM) Gaussian filter. Data acquired during the overlapping movie segments were discarded resulting in a total of 2662 TRs with 1326 TRs in the first session and 1336 TRs in the second session. We derived a gray matter mask by segmenting the MNI_avg152T1 brain provided in AFNI and removing any voxel that was outside the cortical surface by more than twice the thickness of the gray matter at each surface node. It included 54,034 3 mm isotropic voxels across both hemispheres (Guntupalli et al., 2016).

Permutations for Identity and Familiarity decoding

Statistical significance of the decoding analyses was computed using permutation testing (Stelzer, Chen, & Turner, 2013) coupled with Threshold-Free Cluster Enhancement (Smith & Nichols, 2009), as implemented in CoSMoMVPA (Oosterhof, Connolly, & Haxby, 2016). The identity decoding analysis was performed separately for Familiar and Unfamiliar identities, and permuted datasets were obtained by randomly permuting the four identity labels separately within each of the 11 runs. Out of the $(4!)^{11}$ possible permuted maps, we randomly selected 20 for each subject, and bootstrapped them to generate 10,000 group-level permuted maps (see Methods in main text, and (Stelzer et al., 2013).

For the familiarity decoding, the cross-validation scheme was performed across identities and not across runs. Because each identity had a clear association with a superordinate category that indicated the familiarity of the stimulus (Familiar or Unfamiliar), during permutation it was important not to divide the identity labels across runs into two different familiarity groups. Thus, permutation had to be performed at the level of identities rather than at the superordinate level of familiarity to avoid a possible positive bias. Because the decoding problem was binary, the number of valid

permutations within each subject was much lower than in the identity decoding. For example, consider the eight labels $f_1, f_2, f_3, f_4, u_1, u_2, u_3, u_4$ where f_i indicates familiar identities, and u_i indicates unfamiliar identities; any permutation that maps all familiar identities to unfamiliar identities, such as

$$f_i \rightarrow u_j \forall\ i = \pi(1 \ldots 4), j = \pi'(1 \ldots 4),$$

where π, π' indicate any permutation of the integers 1 to 4, will result in the same accuracy maps as the original one, because the order of the class “Familiar” and “Unfamiliar” does not matter. Thus, at least one identity label within each class must be kept fixed. Because also the order of the identity labels within each class does not matter, without loss of generality one can consider the first familiar identity label f_1 as fixed, with the remaining seven identity labels to be determined. Once three labels have been determined, the remaining four are automatically determined (because the order within class does not matter). Thus, the total number of distinct permutations for this binary classification C_3^7 (7 choose 3). We note that the identity permutation is part of this set of possible permutations. Thus, we performed the familiarity decoding on these 35 permutations, and proceeded as described before to obtain null distribution maps.

Early visual cortex ROI analysis and HMAX features

To further analyze the results in early visual cortex found while decoding familiarity, we performed additional analysis on neural data, selecting early visual area masks from the probabilistic atlas by (Wang, Mruczek, Arcaro, & Kastner, 2015), as well as on visual features extracted from the face images using the HMAX model (Riesenhuber & Poggio, 1999; Serre, Wolf, Bileschi, Riesenhuber, & Poggio, 2007). All decoding analyses were performed with a linear SVM classifier, similar to the analyses in the main text.

For the neural data, we selected the bilateral V1v, V1d, V2v, V2d, V3v, V3d masks, downsampled to the MNI 2mm template, and created eight masks: V1v, V1d, V1; V2v, V2d, V2; V3v, V3d, V3; V1+V2; V1+V2+V3. We selected the voxels in these masks, and for each ROI we performed Familiarity Decoding analysis as follows. We cross-validated across identities, as described in the methods in the main text. Within each training fold, we performed feature selection by keeping only the top 10% voxels according to the F-value of a one-way ANOVA. Such feature selection was performed in each training fold. To perform statistical assessment, we permuted the labels (as described in the methods of the main text) and performed the same analysis, obtaining 35 permuted accuracy values for each ROI and subject. Then, to bootstrap population level statistic, we randomly selected one accuracy value for each subject and each ROI, averaged to obtain an average accuracy across subject, and repeated this process 10,000 times to produce a null distribution of accuracy values (Stelzer et al., 2013). The empirical p-value was then computed as the number of null-distribution

accuracies higher than the original accuracy divided by the total number of bootstraps. We added 1 to both the numerator and the denominator to account for the original value being considered (Ojala & Garriga, 2010). Supplementary Figure 7 shows the results of this analysis.

For the HMAX model features, we used the pre-trained model obtained from http://maxlab.neuro.georgetown.edu/hmax.html, and extracted C1 and C2 features for all the images used in the experiment, stacking them across patch sizes and orientations. Images were resized to 200 x 200 pixels to reduce computation time. For each subject separately (using only the images each subject saw in the experiment), we performed a classification analysis on Familiar vs. Unfamiliar face images across identities, and obtained 33 accuracies (one for each subject). We then generated a null distribution of accuracy values at the group level with the same process used with neural data (see above). Supplementary Figure 8 shows the result of this analysis.

Quantification of similarities within and between core and extended systems

To further quantify the similarity of representations within the systems, we compared the average within-system correlations with the average between-system correlations. We first considered only the areas belonging to the core and extended system (without EV areas). Then, for each one of the distance matrices (corresponding to each subject for the task data, and to each pair of subjects for the movie data), we removed the diagonal and averaged the cells corresponding to the within-system correlations (core or extended system ROIs) and to the between-system correlations (core system ROIs correlated to extended system ROIs), and computed the difference between these two values. These differences were bootstrapped 10,000 times to obtain 95% BCa (bias-corrected and accelerated) confidence intervals (DiCiccio & Efron, 1996). This process was repeated for the further subdivisions of the core system (anterior, dorsal, ventral core system) and extended system (theory of mind, precuneus), considering only ROIs belonging to the core and extended system respectively: for example, the between-system correlations for the anterior core system were considered to be only the correlations between the anterior core ROIs with the dorsal and ventral core ROIs.

Comparison of second-order representational geometries obtained from task data and movie data

We quantified the degree of similarity of the distance matrices used to generate the two MDS plots (one for the task data, and one for the movie data) by correlating (Spearman correlation) the upper triangular matrices of the distances, as well as computing the RV-coefficient, a measure of similarity between square matrices (Abdi, 2007, 2010; Robert & Escoufier, 1976). Before computing the RV-coefficient, the

distance matrices were normalized as follows (Abdi, Williams, Valentin, & Bennani-Dosse, 2012). Given a distance matrix *D*, we computed a cross-product matrix $\hat{S} = -\frac{1}{2}CDC^T$, where $C = I - \frac{1}{n}O$ is the centering matrix, and *O* is a n x n matrix of ones. Then, $\hat{S}$ was normalized by its first eigenvalue, resulting in $S = \hat{S}/\lambda_1$. We then computed the RV-coefficient between the two normalized distance matrices. To obtain 95% confidence intervals, we bootstrapped the individual subjects' distance matrices for the task data, and computed both the correlation and the RV-coefficient against the movie distance matrix.

To visualize the match between the MDS solutions obtained from the two datasets, we performed Procrustes alignment to obtain an affine transformation that would align the movie solution to the task solution, and visualized the two solutions in the first two dimensions (see Supplementary Figure 13).

Model-based RSA within systems

We performed model-based RSA within each ROI and for each subject separately using the task data. For each ROI we computed a neural RDM and correlated it (Spearman partial correlation) with four target model RDMs: a familiarity RDM, an identity RDM, and two RDMs obtained from the C1 and C2 layers of the HMAX model (Riesenhuber & Poggio, 1999) to control for low-level visual differences. The correlations were then averaged across the ROIs in each system, and visualized in Supplementary Figure 15.

Distances of EV ROIs from dorsal and ventral systems

We analyzed the position of the EV with respect to the ventral and dorsal streams by computing the following "Dorsal" index

$$I = \frac{d(\text{ventral}, \text{EV}) - d(\text{dorsal}, \text{EV})}{d(\text{ventral}, \text{EV}) + d(\text{dorsal}, \text{EV})}$$

where *d(ventral, EV)* and *d(dorsal, EV)* indicate the average distance to the EV of the ventral and dorsal stream respectively; a positive index indicates that the EV is farther from the ventral stream, while a negative index indicates that the EV is closer to the ventral stream. We performed this analysis separately for each hemisphere and for the four EV ROIs using the task data to allow for statistical testing, before any dimensionality reduction with MDS.

Supplementary Tables

Table 1. MNI coordinates of the ROI centers sorted and color-coded with respect to the system they belong to, according to the models of (Duchaine & Yovel, 2015; Gobbini & Haxby, 2007; Guntupalli, Wheeler, & Gobbini, 2017; Haxby & Gobbini, 2011; Haxby, Hoffman, & Gobbini, 2000). Abbreviation in parenthesis refers to the statistical map that was used to individuate the ROI: FAM, familiarity decoding; ID, identity decoding (see Methods in the main text for details). See also http://neurovault.org/collections/NEUNABLT/images/46823/ for the position of the ROIs.

ROI	Abbreviation	x	y	z
**Early visual**				
early visual - left (ID)	EV1 - L	-4	-86	4
early visual - right (ID)	EV1 - R	10	-94	4
early visual - left (FAM)	EV2 - L	-4	-86	-6
early visual - right (FAM)	EV2 - R	4	-90	-10
**Core face system (Haxby et al. 2000)**				
**Ventral core system**				
Occipital fusiform – left (ID)	OccFus - L	-32	-80	-14
Occipital fusiform – right (ID)	OccFus - R	22	-80	-12
Posterior temporal fusiform – left (ID)	pFus - L	-36	-60	-14
Posterior temporal fusiform – right (ID)	pFus - R	28	-72	-14
Mid temporal fusiform – left (ID)	mFus - L	-34	-44	-18
Mid temporal fusiform – right (FAM)	mFus - R	38	-38	-24
Anterior temporal fusiform – right (FAM)	aFus - R	40	-14	-28
**Dorsal core system**				
Anterior middle temporal gyrus – left (FAM)	pMTG - L	-66	-48	4
Anterior middle temporal gyrus – right (FAM)	pMTG - R	62	-44	0
Mid middle temporal gyrus – left (FAM)	mMTG - L	-64	-30	-8
Mid middle temporal gyrus – right (FAM)	mMTG - R	60	-34	-2
Anterior middle temporal gyrus – left (FAM)	aMTG - L	-58	0	-24
Anterior middle temporal gyrus – right (FAM)	aMTG - R	62	-14	-12
**Anterior core system**				
Inferior frontal gyrus – left (ID)	IFG1 - L	-50	40	-8
Inferior frontal gyrus - left (FAM)	IFG2 - L	-38	46	2

Inferior frontal gyrus – right (FAM)	IFG2 - R	46	26	-2
**Extended face system**				
**Theory of mind**				
Temporoparietal junction – left (FAM)	TPJ - L	-58	-58	28
Temporoparietal junction – right (FAM)	TPJ - R	50	-54	30
Medial prefrontal cortex – left (FAM)	MPFC - L	-8	48	-8
Medial prefrontal cortex – right (FAM)	MPFC - R	14	44	12
**Precuneus**				
Dorsal precuneus - left (ID)	dPreCun - L	-6	-66	50
Dorsal precuneus - right (ID)	dPreCun - R	6	-64	50
Mid precuneus – left (FAM)	mPreCun - L	-10	-56	34
Mid precuneus – right (FAM)	mPreCun - R	4	-66	32
Ventral precuneus – left (FAM)	vPreCun - L	-8	-54	20
Ventral precuneus – right (FAM)	vPreCun - R	8	-54	22

Table 2. Parameter estimates for the Linear Mixed-Effect model on ROI correlations from the task data. 95% confidence intervals were computed through parametric bootstrapping (see main text for Methods). Labels in bold show estimates significantly different from 0.

**Parameter**	**Estimate**	**SE**	**t-value**	**Left CI**	**Right CI**
**Intercept (β)**	**0.0644**	0.0065	9.9535	0.0518	0.0771
Within Core vs. Between (β)	0.0044	0.0043	1.0033	-0.0041	0.0128
**Within Extended vs. Between (β)**	**0.0993**	0.0061	16.3607	0.0869	0.1111
**sigma (subjects)**	**0.0334**			0.0241	0.0423
**sigma (residuals)**	**0.2066**			0.2039	0.2094

Table 3. Parameter estimates for the Linear Mixed-Effect model on ROI correlations from the movie data. 95% confidence intervals were computed through parametric bootstrapping (see main text for Methods). Labels in bold show estimates significantly different from 0.

**Parameter**	**Estimate**	**SE**	**t-value**	**Left CI**	**Right CI**
**Intercept (β)**	**0.4719**	0.0040	116.8884	0.4641	0.4797
**Within Core vs. Between (β)**	**0.0678**	0.0030	22.4696	0.0619	0.0738
**Within Extended vs. Between (β)**	**0.1479**	0.0042	35.0654	0.1398	0.1565
**sigma (subjects)**	**0.0261**			0.0204	0.0318
**sigma (residuals)**	**0.1854**			0.1835	0.1874

Table 4. Difference of correlations for within- and between-system ROIs with the task data. Correlations of the subsystems of core and extended areas were computed using only ROIs for core and extended system respectively. Labels and differences in bold show systems whose 95% bootstrapped confidence intervals did not contain 0.

System	Within	Between	Difference	Left CI	Right CI
Core	0.0687	0.0644	0.0044	-0.0086	0.0177
**Extended**	0.1637	0.0644	**0.0993**	0.0846	0.1153
Ventral Core	0.0815	0.0660	0.0154	-0.0045	0.0360
**Dorsal Core**	0.0966	0.0643	**0.0323**	0.0135	0.0520
Anterior Core	0.0573	0.0549	0.0024	-0.0349	0.0393
Precuneus	0.0934	0.0952	-0.0018	-0.0252	0.0227
Theory of Mind	0.1054	0.0952	0.0102	-0.0198	0.0427

Table 5. Difference of correlations for within- and between-system ROIs with the movie data. Correlations of the subsystems of core and extended areas were computed using only ROIs for core and extended system respectively. Labels and differences in bold show systems whose 95% bootstrapped confidence intervals did not contain 0.

System	Within	Between	Difference	Left CI	Right CI
**Core**	0.5397	0.4719	**0.0678**	0.0633	0.0720
**Extended**	0.6198	0.4719	**0.1479**	0.1427	0.1533
**Ventral Core**	0.5459	0.3961	**0.1498**	0.1424	0.1567
**Dorsal Core**	0.8518	0.4884	**0.3635**	0.3508	0.3749
**Anterior Core**	0.8025	0.5636	**0.2389**	0.2277	0.2513
**Precuneus**	0.7268	0.5541	**0.1727**	0.1631	0.1808
**Theory of Mind**	0.6154	0.5541	**0.0613**	0.0510	0.0730

Supplementary Figures

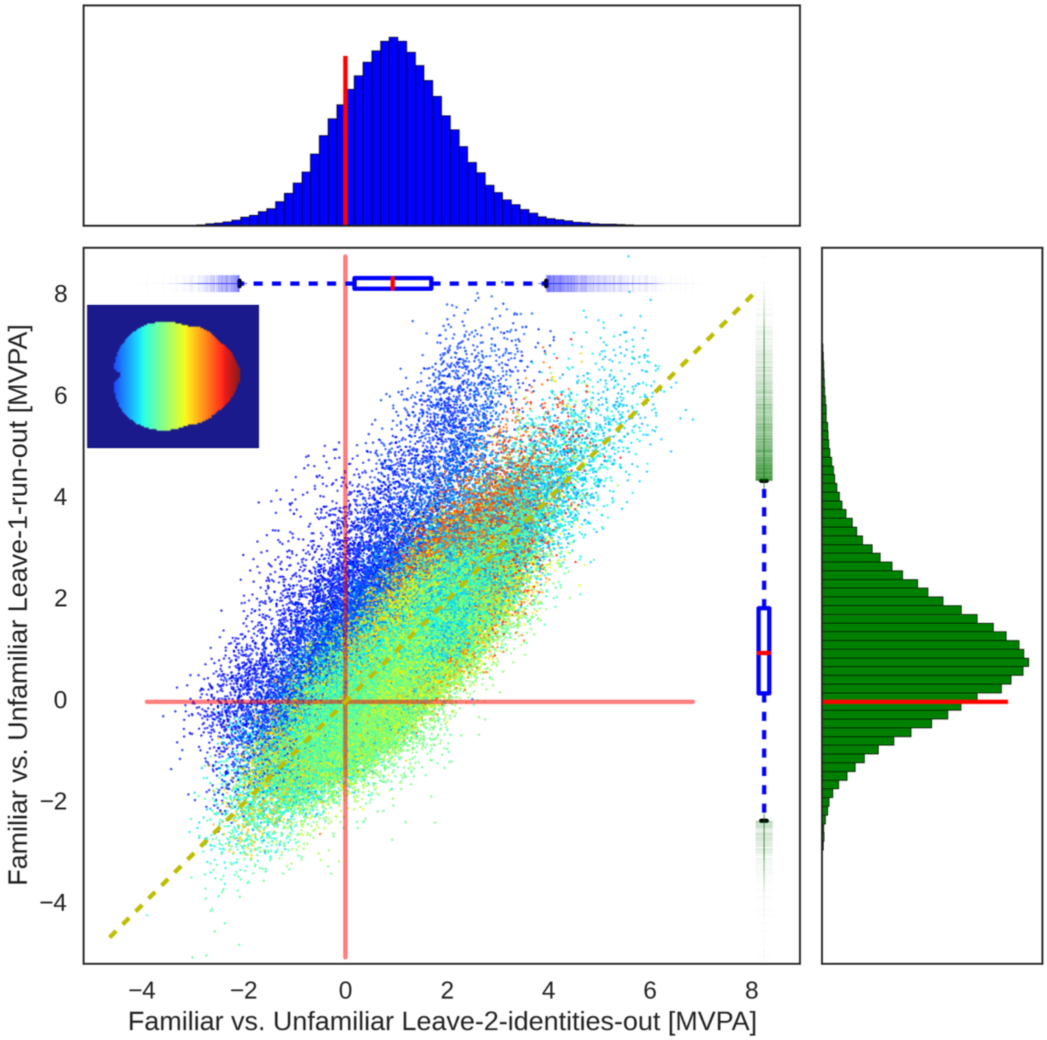

Figure 1. Comparison of cross-validation schemes for the familiarity decoding. The x-axis shows z-values from the familiarity classification using the leave-2-identities-out scheme, as reported in the main manuscript. The y-axis shows the same classification using a common leave-one-run-out scheme. Colors of the points map the location of the voxel in the axial plane, as shown in the inset. The leave-2-identities-out scheme successfully controls for identity information, as can be seen by the overall lower z-values for voxels belonging to the occipital cortex. The maps used to generate this plot are http://neurovault.org/collections/NEUNABLT/images/46809/ (x-axis) and http://neurovault.org/collections/NEUNABLT/images/46812/ (y-axis).

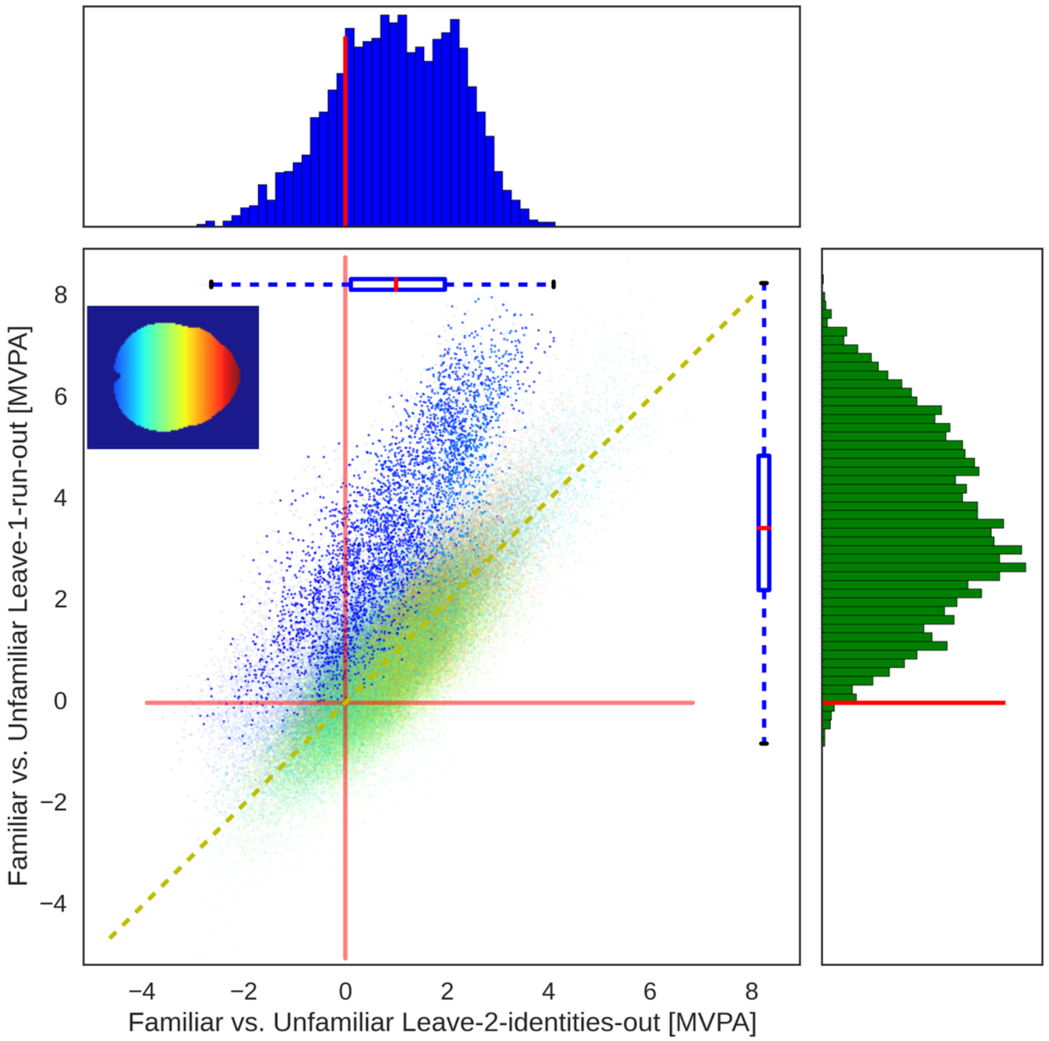

Figure 2. Comparison of cross-validation schemes for the familiarity decoding, highlighting only voxels belonging to a probabilistic EV mask (V1 + V2 + V3) (Wang et al., 2015)**.** The x-axis shows z-values from the familiarity classification using the leave-2-identities-out scheme, as reported in the main manuscript. The y-axis shows the same classification using a common leave-one-run-out scheme. Colors of the points map the location of the voxel in the axial plane, as shown in the inset. The leave-2-identities-out scheme successfully controls for visual information, as can be seen by the overall lower z-values for voxels belonging to the early visual areas. The maps used to generate this plot are http://neurovault.org/collections/NEUNABLT/images/46809/ (x-axis) and http://neurovault.org/collections/NEUNABLT/images/46812/ (y-axis).

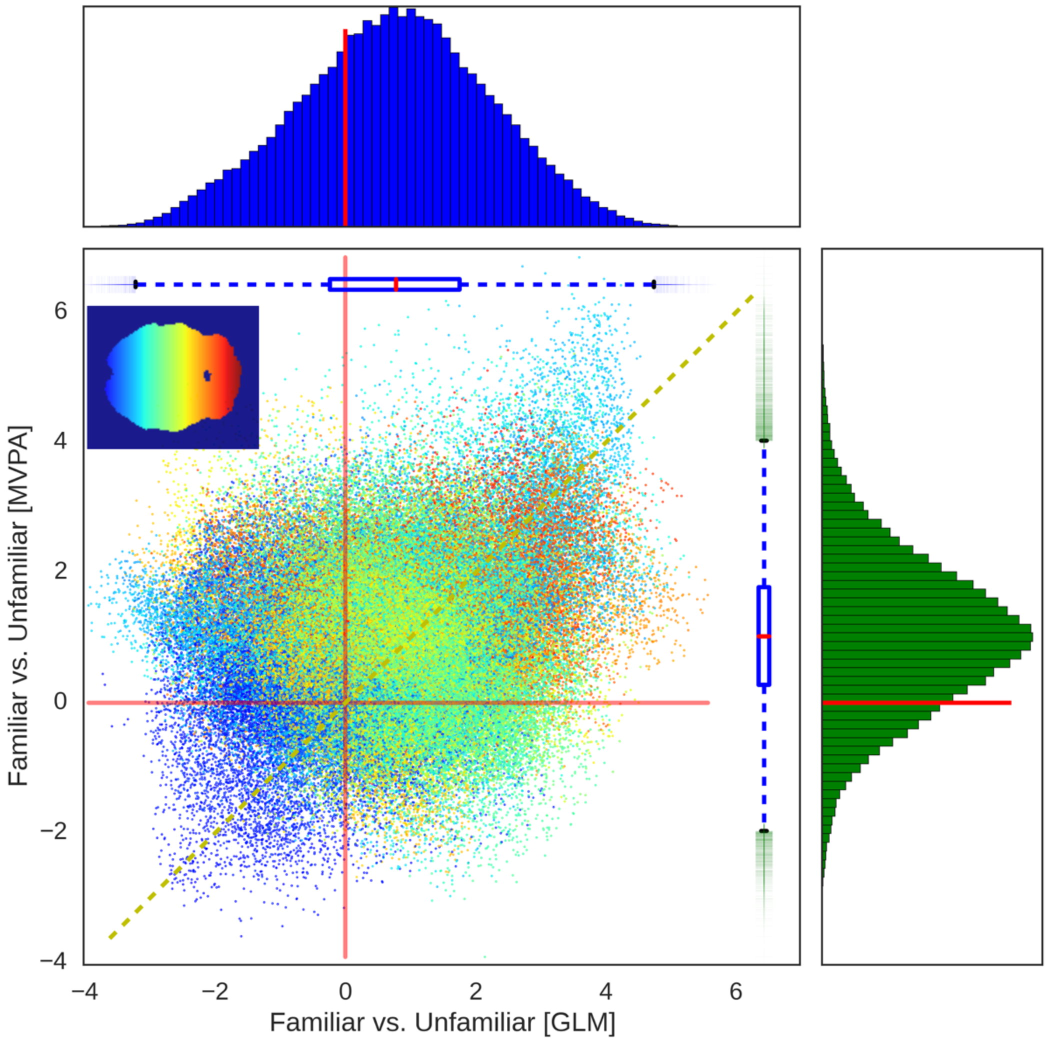

Figure 3. Comparison of the univariate analysis of familiarity with the MVPA familiarity decoding. The x-axis shows z-values from the univariate contrast Familiar > Unfamiliar. The y-axis shows the z-values of the Familiarity classification across identities. Colors of the points map the location of the voxel in the axial plane, as shown in the inset. The maps used to generate this plot are http://neurovault.org/collections/NEUNABLT/images/46809/ (x-axis) and http://neurovault.org/collections/NEUNABLT/images/46813/ (y-axis).

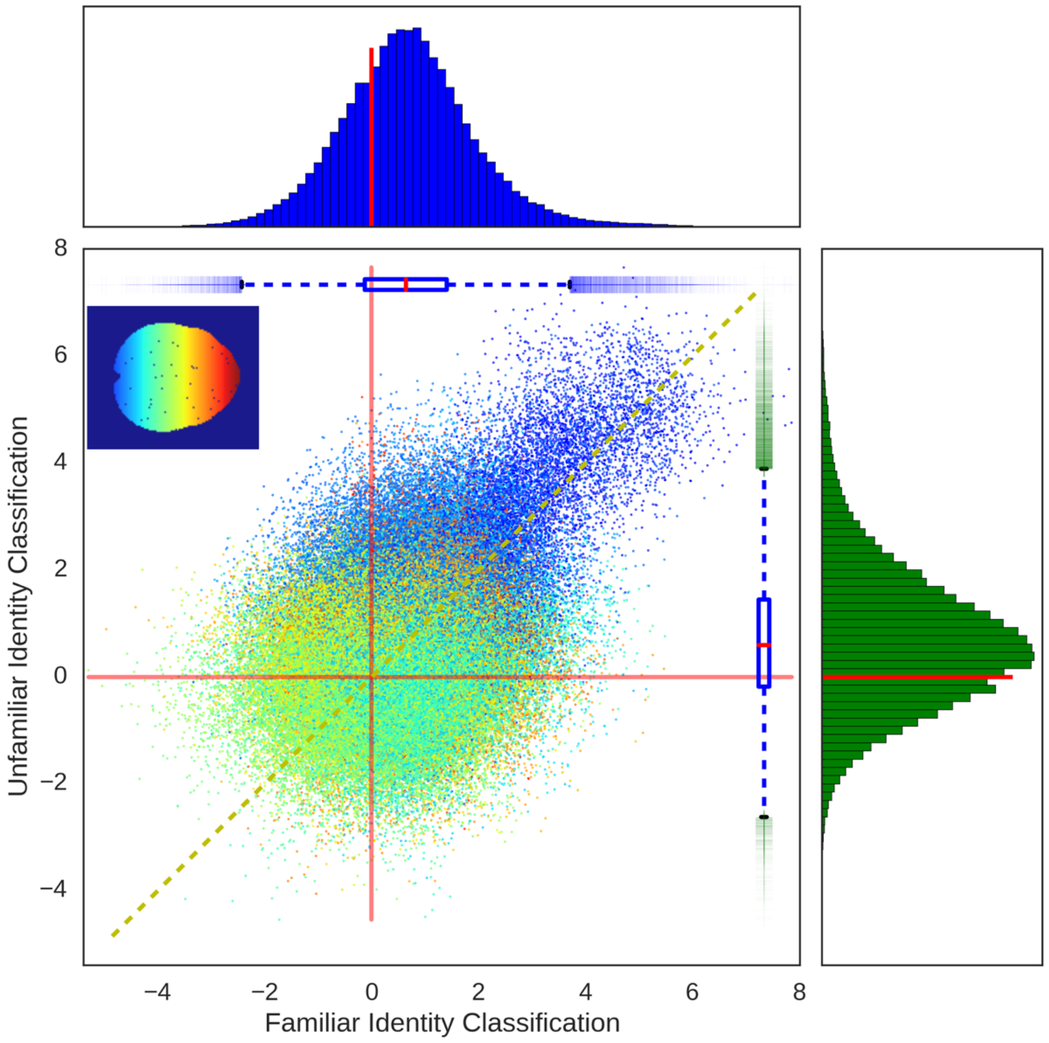

Figure 4. Comparison of the MVPA identity decoding within familiar identities vs. within unfamiliar identities. The x-axis shows z-values of the Familiar Identity Classification (see Supplementary Figure 5 for a thresholded map). The y-axis shows the z-values of Unfamiliar Identity Classification (see Supplementary Figure 6 for a thresholded map). Colors of the points map the location of the voxel in the axial plane, as shown in the inset. The maps used to generate this plot are http://neurovault.org/collections/NEUNABLT/images/46817/ (x-axis) and http://neurovault.org/collections/NEUNABLT/images/46820/ (y-axis).

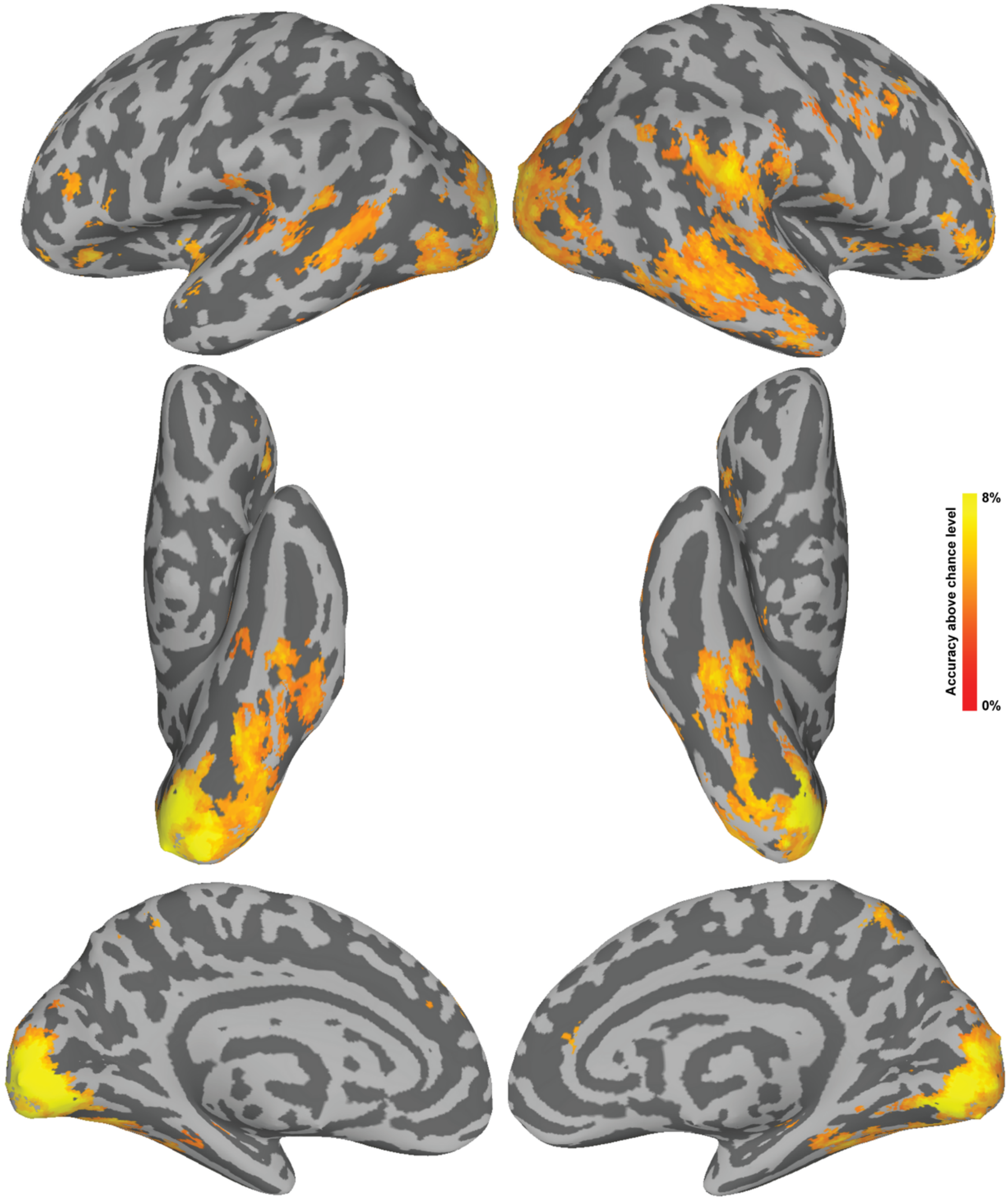

Figure 5. Searchlight maps for the Identity classification with familiar identities only. Maps were thresholded at a z-TFCE score of 1.65, corresponding to $p < 0.05$ one-tailed (corrected for multiple comparisons).

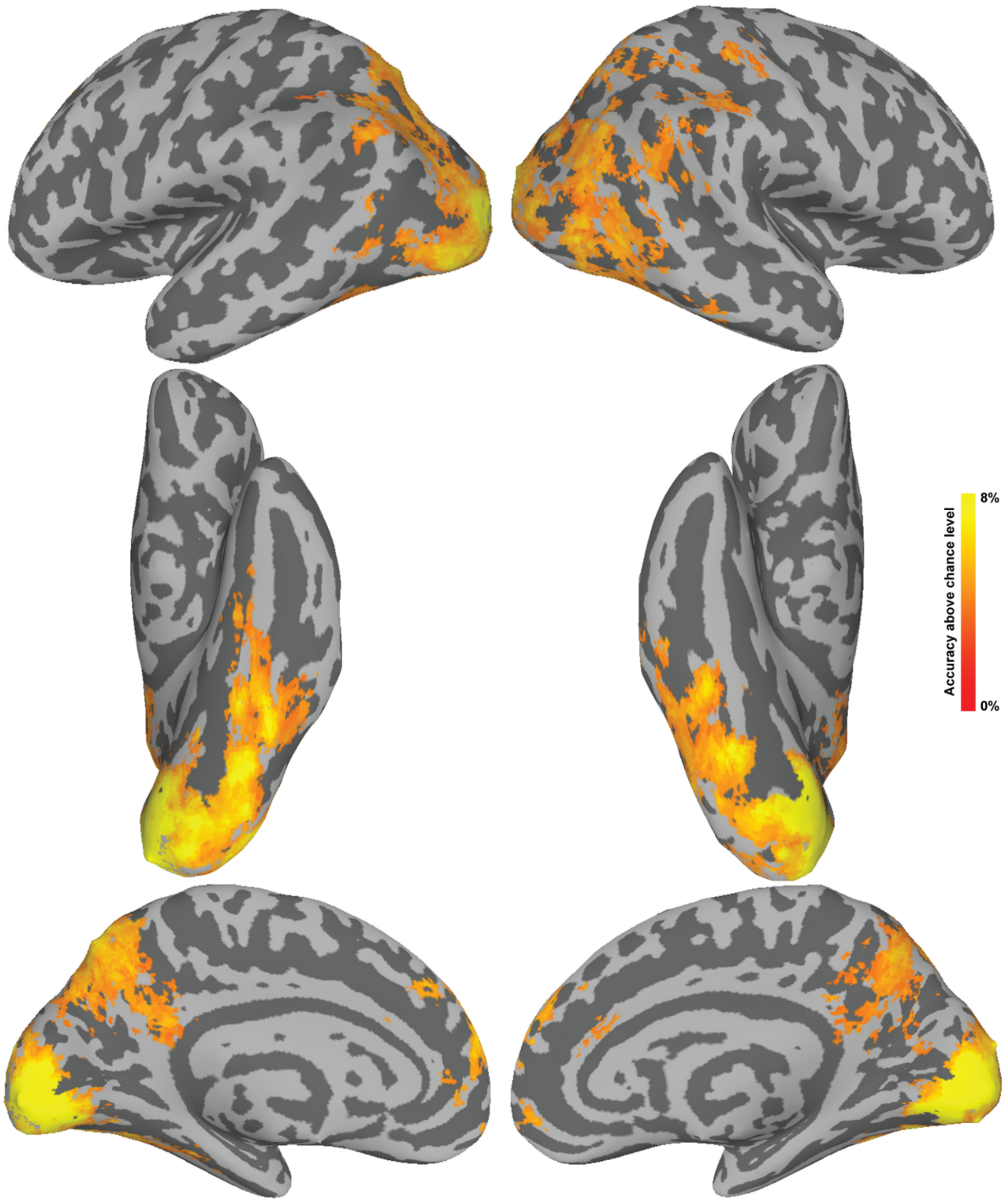

Figure 6. Searchlight maps for the Identity classification with unfamiliar identities only. Maps were thresholded at a z-TFCE score of 1.65, corresponding to $p < 0.05$ one-tailed (corrected for multiple comparisons).

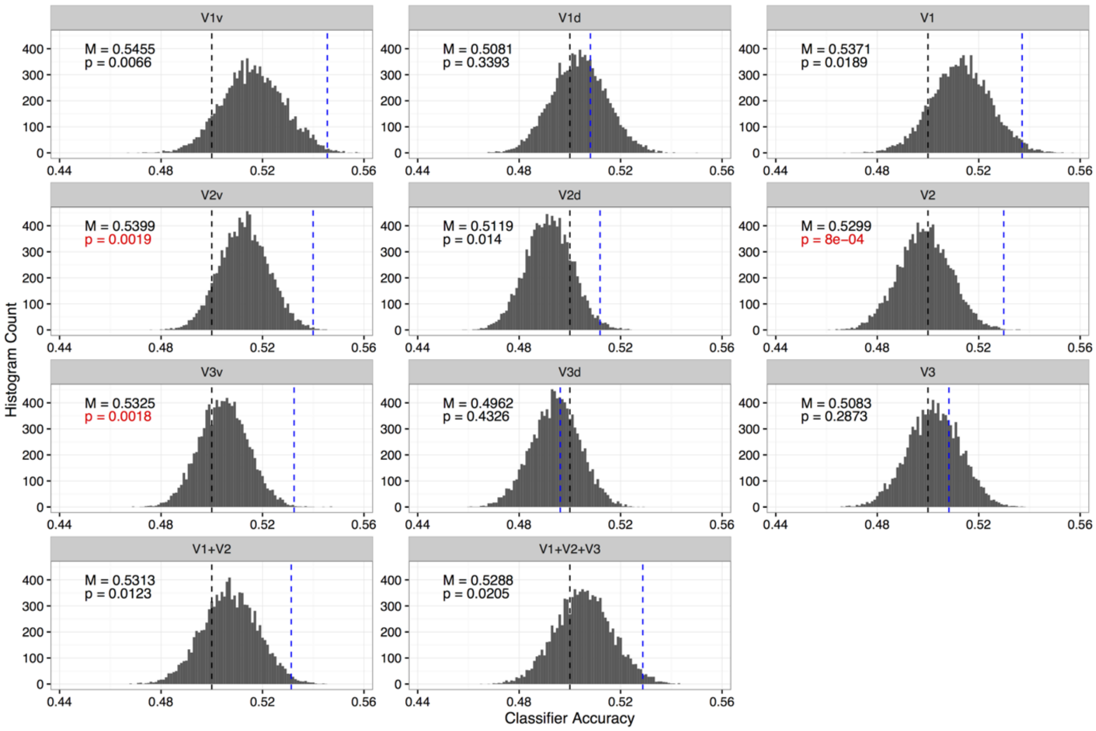

Figure 7. Familiarity decoding across identities in early visual cortex ROIs. Probabilistics masks were obtained from (Wang et al., 2015), see Supplementary Methods. Histograms show the null distribution obtained by permuting labels. Dashed black line shows 50% accuracy. Dashed blue line shows the average accuracy across participants in the original (non-permuted) dataset. Red p-values indicated significance at p = 0.05, Bonferroni corrected.

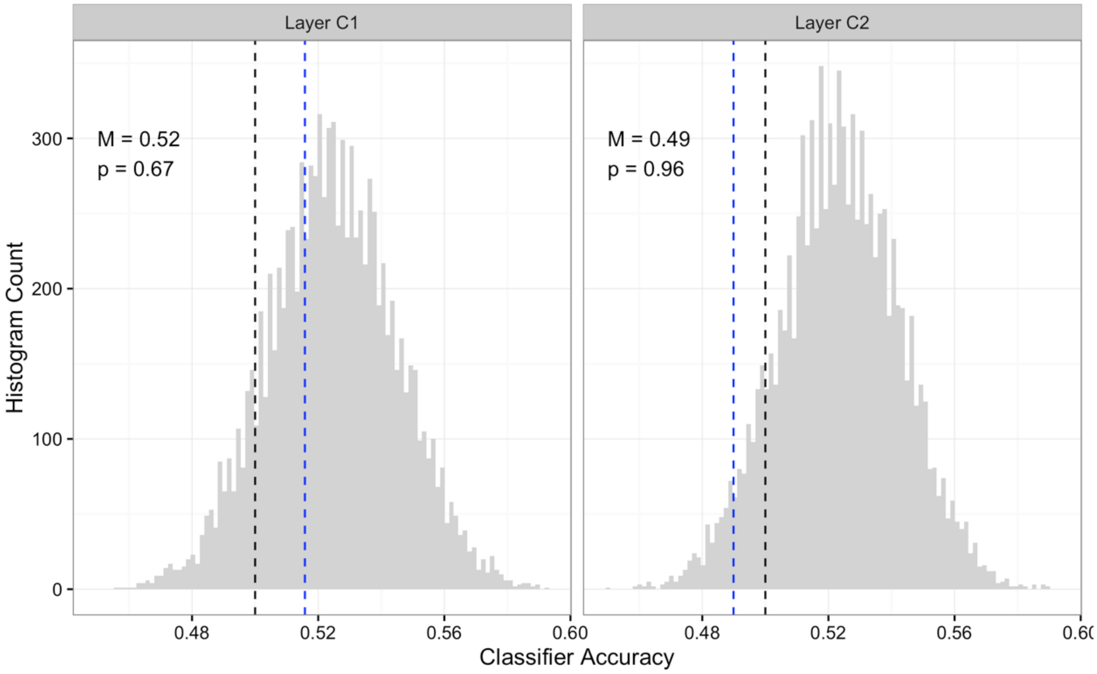

Figure 8. Familiarity decoding across identities using features extracted from the HMAX model. Features extracted in Layer C1 and C2 from the HMAX model (Riesenhuber & Poggio, 1999; Serre et al., 2007) were used to classify the images used in the experiment, using a leave-two-identities-out cross-validation scheme (see Supplementary Methods and Methods in main text). Dashed black line shows 50% accuracy. Dashed blue line shows the average accuracy across participants in the original (non-permuted) dataset (indicated as M in the inset).

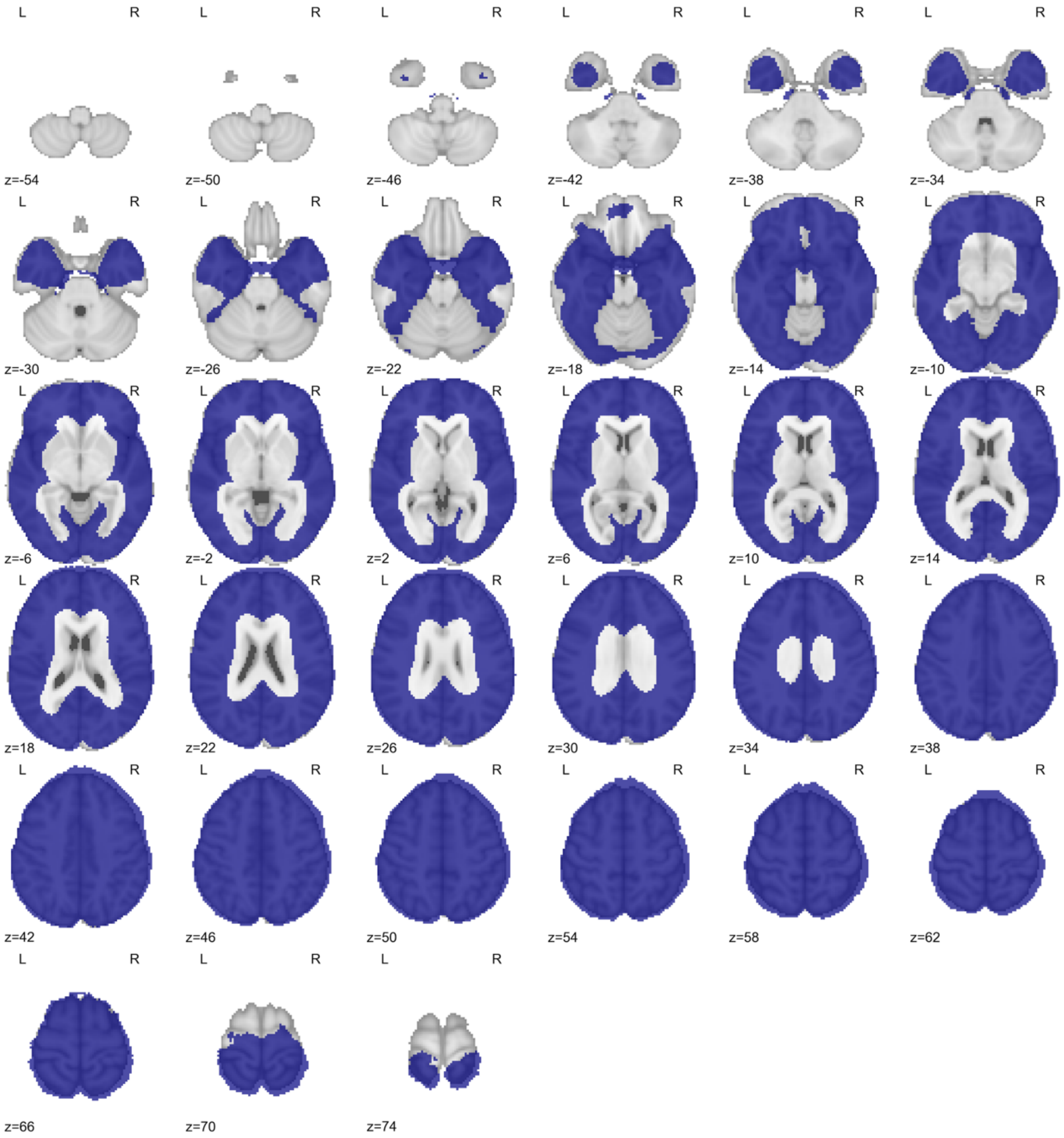

Figure 9. Mask used in the multivariate analyses. See http://neurovault.org/collections/NEUNABLT/images/46822/.

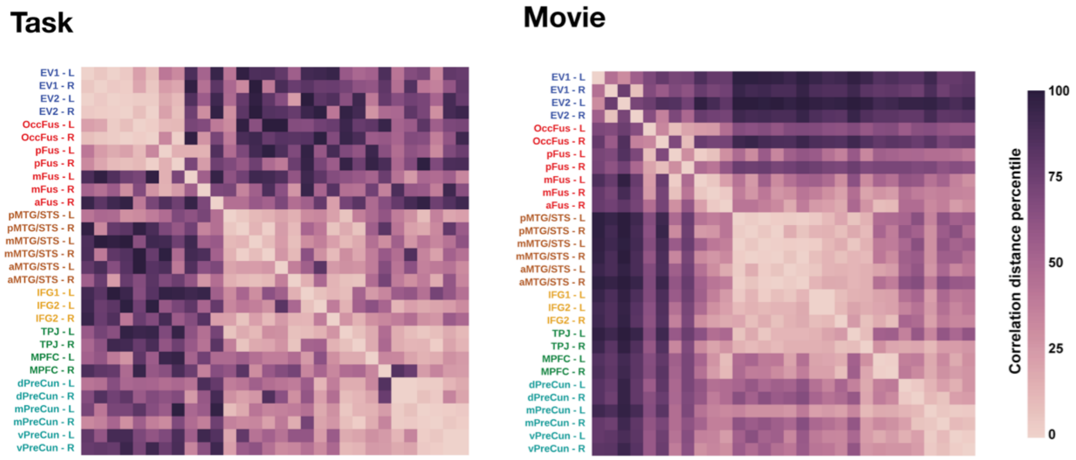

Figure 10. Distance matrices between pairwise RDMs in each ROI for the task data (left) and the movie data (right). Colors represent correlation distance percentile for display purposes, with lighter colors indicating higher similarity and darker colors lower similarity.

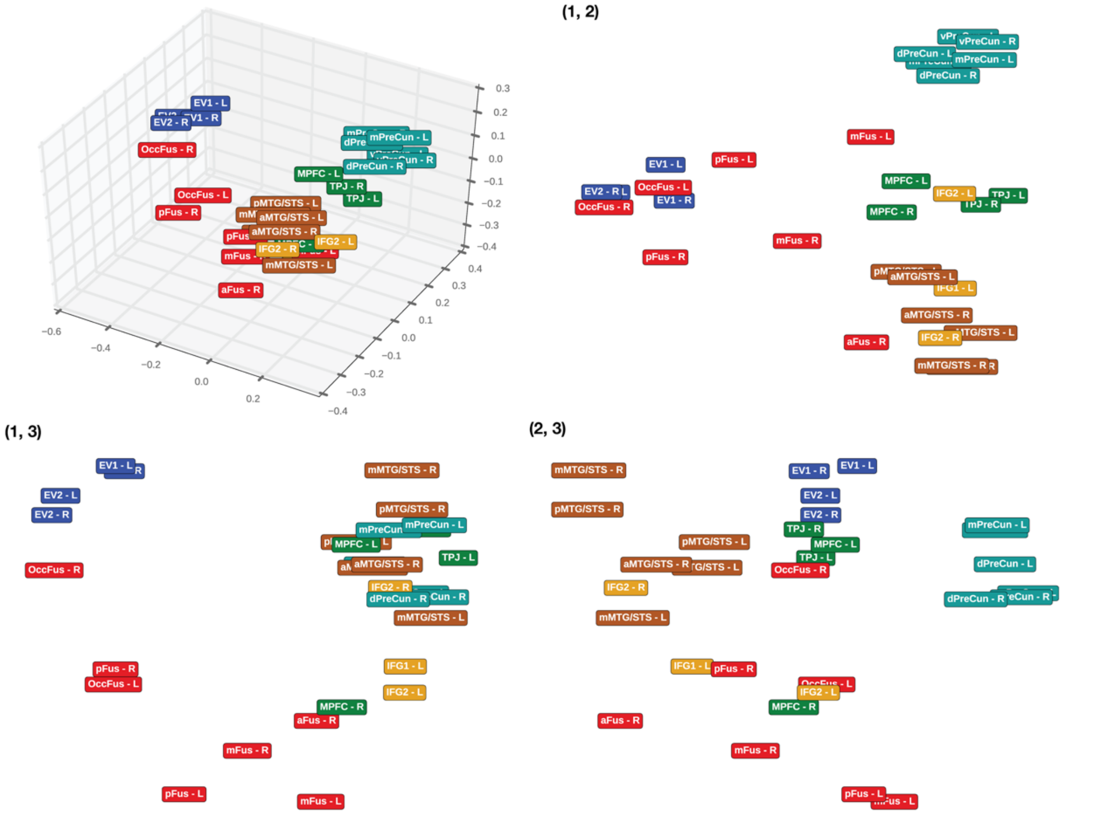

Figure 11. Three-dimensional MDS solution computed on the task data, with projections on each pair of dimensions. Top left panel shows full 3D solution, top right panel shows the projection on the first and second dimension, bottom left on the first and third dimension, and bottom right on the second and third dimension. Because the MDS solution is invariant to orientation, the second dimension was multiplied by -1 to have the precuneus ROIs on top. In the figure in the main text labels were minimally jittered to avoid overlaps and increase legibility; here we report here the original non-jittered position.

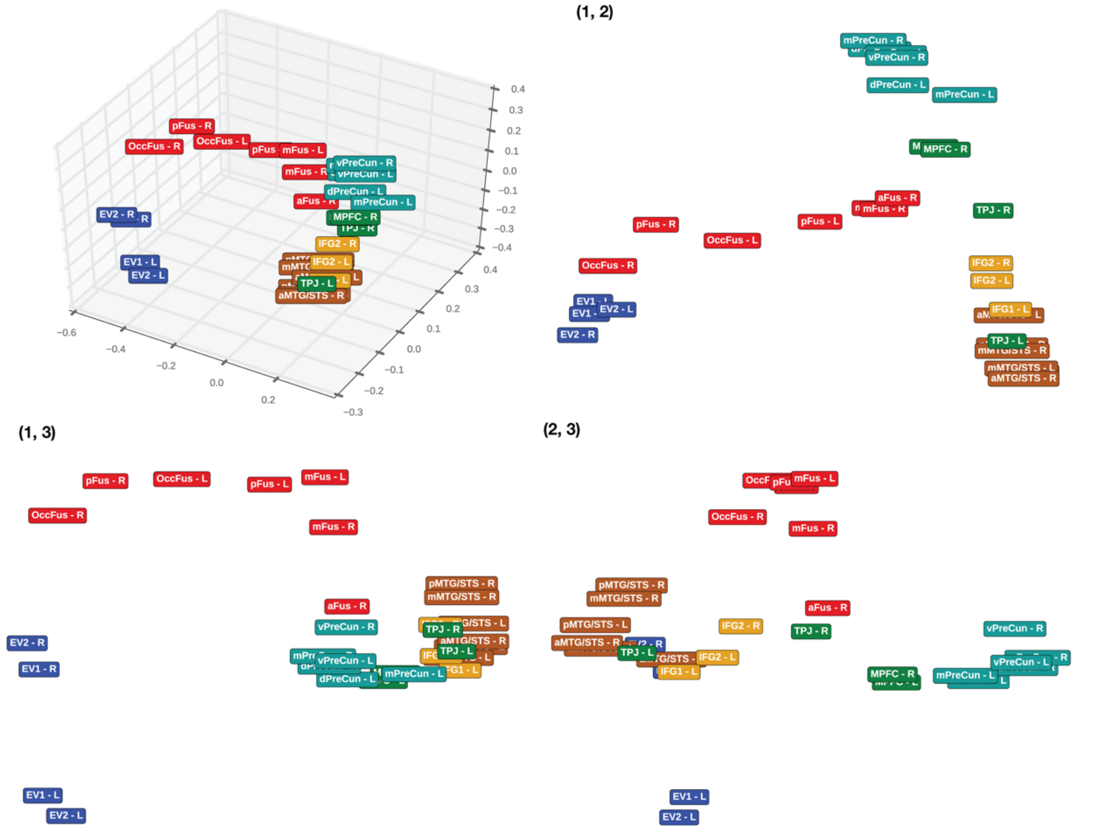

Figure 12. Three-dimensional MDS solution computed on the hyperaligned movie data, with projections on each pair of dimensions. Top left panel shows full 3D solution, top right panel shows the projection on the first and second dimensions, bottom left on the first and third dimensions, and bottom right on the second and third dimensions. In the figure in the main text labels were minimally jittered to avoid overlaps and increase legibility; here we report here the original non-jittered position.

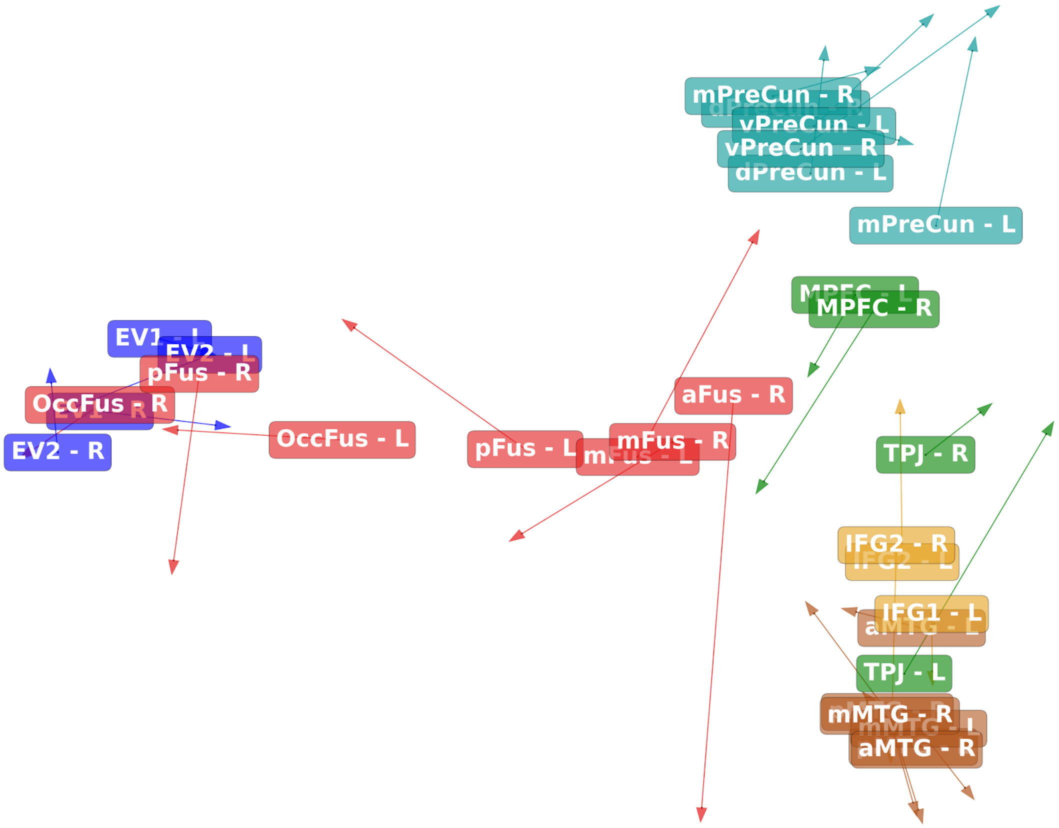

Figure 13. Comparison between the MDS solution of the movie data and the task data. Labels indicate the solution of the movie data; arrows indicate the change in the task data solution. Given the two 3D MDS solutions, we performed Procrustes alignment to obtain an affine transformation that would align the movie solution to the task solution, and re-plotted the movie MDS solution with arrows indicating how the MDS solution changed in the movie data.

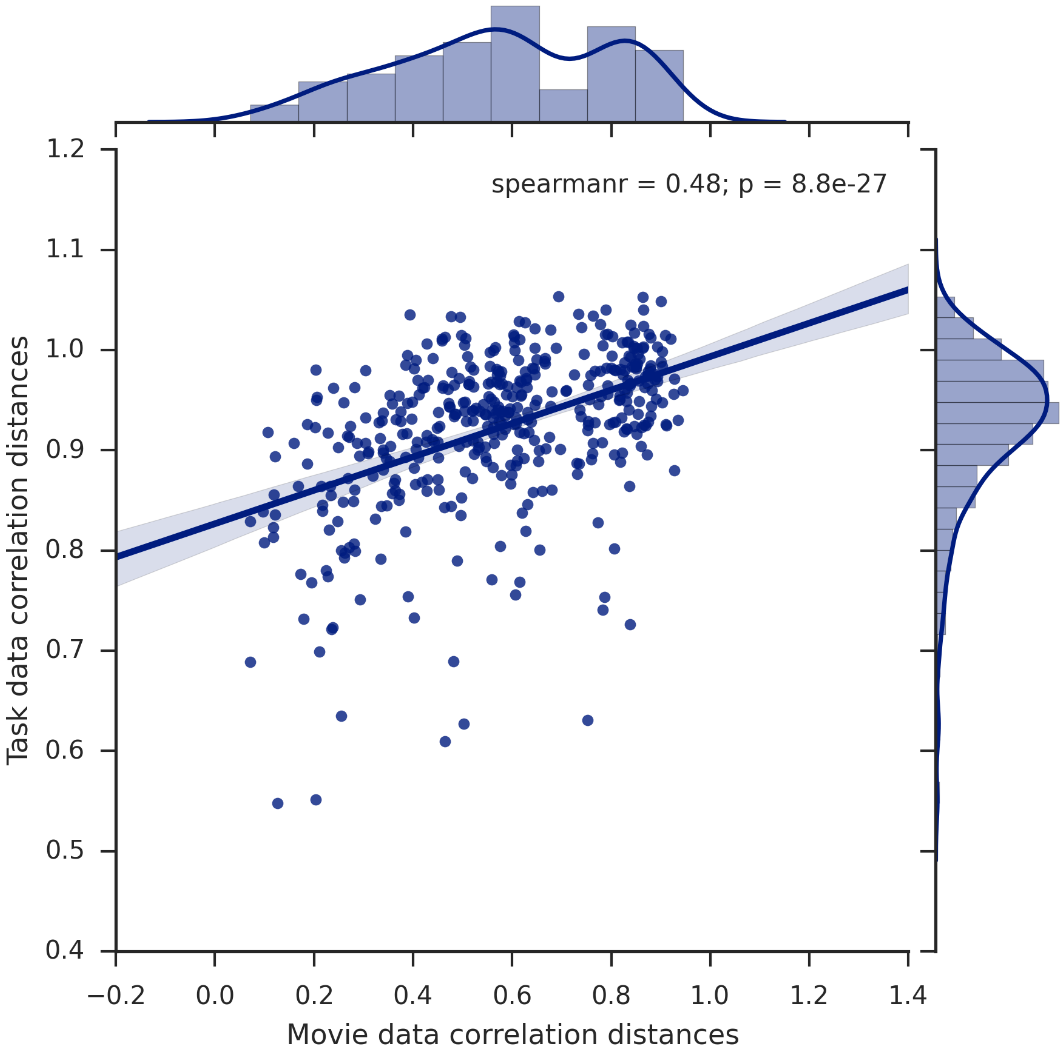

Figure 14. Scatterplot showing the significant correlation of the two distance matrices obtained from the movie data (x-axis) and task data (y-axis). The two distance matrices were significantly similar (RV-coefficient = 0.755 [0.7254, 0.7612]; Spearman r = 0.48 [0.34, 0.49]).

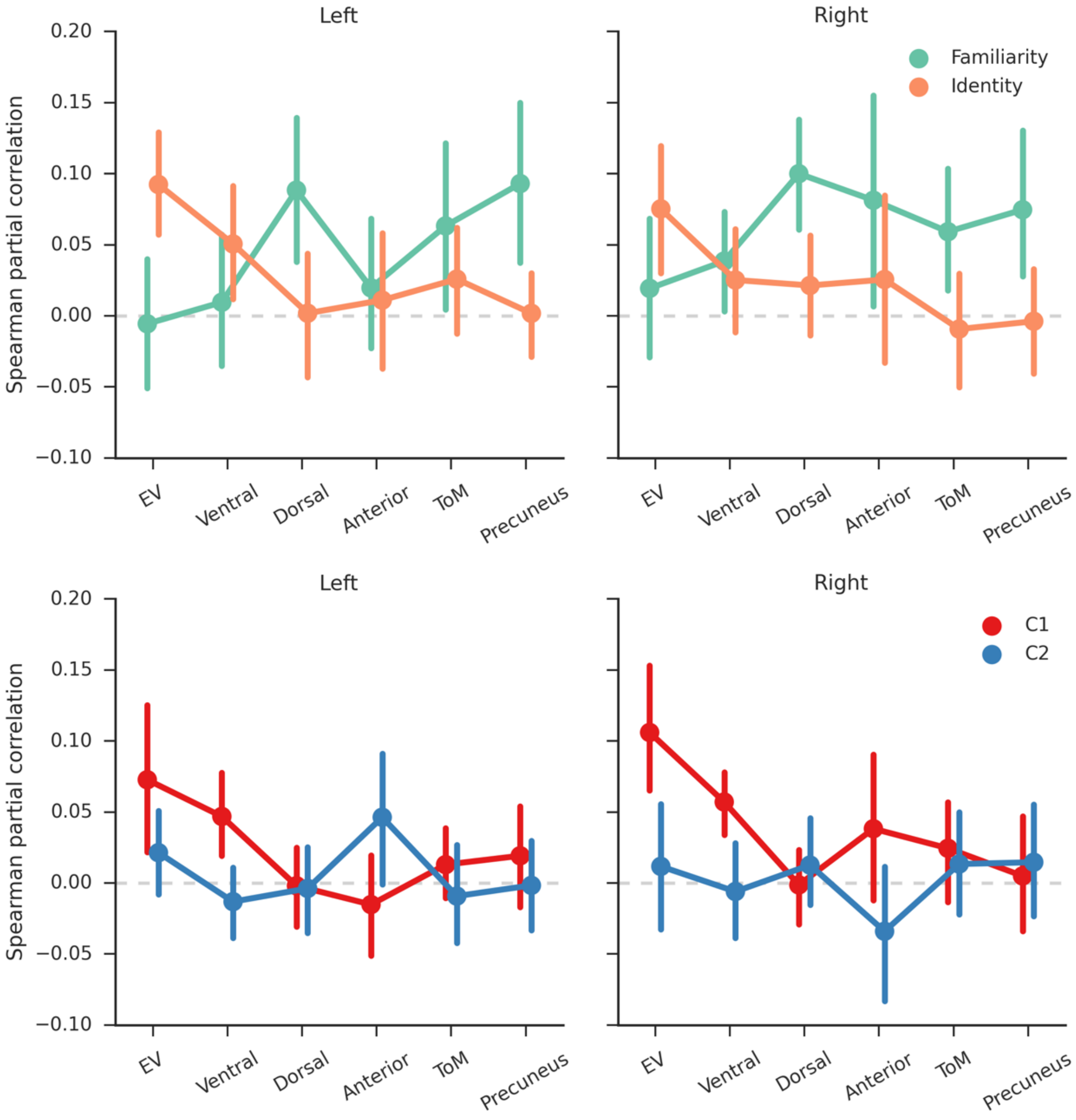

Figure 15. Partial Spearman correlation between the neural RDMs with model RDMs, averaged across the ROIs of each system. The top row shows the results for the familiarity and identity models, while the bottom row shows the results for the models obtained from the C1 and C2 features of the HMAX model (Riesenhuber & Poggio, 1999).

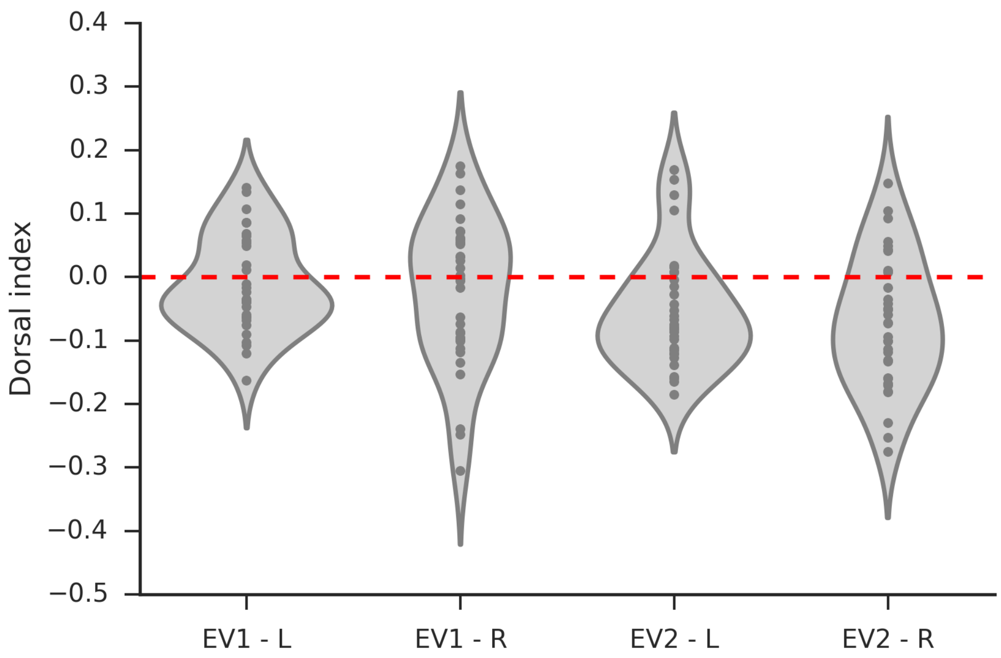

Figure 16. Index of similarity of the EV ROIs to the dorsal system. Each point indicates a single subject's index. Positive values indicate that the EV ROI is closer to the dorsal stream, while negative values indicate that the EV ROI is closer to the ventral stream. EV2 ROIs, obtained from the familiarity decoding map, were slightly but significantly closer to the ventral stream than the dorsal stream: EV2 - L -0.05 [-0.08, -0.02]; EV2 - R -0.07 [-0.11, -0.04] . EV1 ROIs, obtained from the identity decoding map, were equidistant from the dorsal and ventral systems: EV1 - L (-0.01 [-0.04, 0.01]); EV1 - R: -0.02 [-0.07, 0.01].

Supplementary References

Abdi, H. (2007). RV coefficient and congruence coefficient. *Encyclopedia of Measurement and Statistics*, 849–853.

Abdi, H. (2010). Congruence: Congruence coefficient, RV coefficient, and mantel coefficient. *Encyclopedia of Research Design*. Sage, Thousand Oaks, Calif, USA. Retrieved from http://www.utd.edu/~herve/abdi-congruence2010-pretty.pdf

Abdi, H., Williams, L. J., Valentin, D., & Bennani-Dosse, M. (2012). STATIS and DISTATIS: optimum multitable principal component analysis and three way metric multidimensional scaling. *Wiley Interdisciplinary Reviews. Computational Statistics*, *4*(2), 124–167.

DiCiccio, T. J., & Efron, B. (1996). Bootstrap Confidence Intervals. *Statistical Science: A Review Journal of the Institute of Mathematical Statistics*, *11*(3), 189–212.

Duchaine, B., & Yovel, G. (2015). A Revised Neural Framework for Face Processing. *Annual Review of Vision Science*, *1*(1), 393–416.

Gobbini, M. I., & Haxby, J. V. (2007). Neural systems for recognition of familiar faces. *Neuropsychologia*, *45*(1), 32–41.

Guntupalli, J. S., Hanke, M., Halchenko, Y. O., Connolly, A. C., Ramadge, P. J., & Haxby, J. V. (2016). A Model of Representational Spaces in Human Cortex. *Cerebral Cortex* , bhw068.

Guntupalli, J. S., Wheeler, K. G., & Gobbini, M. I. (2017). Disentangling the Representation of Identity from Head View Along the Human Face Processing Pathway. *Cerebral Cortex* . https://doi.org/10.1093/cercor/bhw344

Haxby, J. V., & Gobbini, M. I. (2011). Distributed neural systems for face perception. *Oxford Handbook of Face Perception*, 93.

Haxby, J. V., Guntupalli, J. S., Connolly, A. C., Halchenko, Y. O., Conroy, B. R., Gobbini, M. I., … Ramadge, P. J. (2011). A Common, High-Dimensional Model of the Representational Space in Human Ventral Temporal Cortex. *Neuron*, *72*(2), 404–416.

Haxby, J. V., Hoffman, E. A., & Gobbini, M. I. (2000). The distributed human neural system for face perception. *Trends in Cognitive Sciences*, *4*(6), 223–233.

Ojala, M., & Garriga, G. C. (2010). Permutation Tests for Studying Classifier Performance. *Journal of Machine Learning Research: JMLR*, *11*(Jun), 1833–1863.

Oosterhof, N. N., Connolly, A. C., & Haxby, J. V. (2016). CoSMoMVPA: Multi-Modal Multivariate Pattern Analysis of Neuroimaging Data in Matlab/GNU Octave. *Frontiers in Neuroinformatics*, *10*, 27.

Riesenhuber, M., & Poggio, T. (1999). Hierarchical models of object recognition in cortex. *Nature Neuroscience*, *2*(11), 1019–1025.

Robert, P., & Escoufier, Y. (1976). A Unifying Tool for Linear Multivariate Statistical Methods: The RV- Coefficient. *Journal of the Royal Statistical Society. Series C, Applied Statistics*, *25*(3), 257–265.

Serre, T., Wolf, L., Bileschi, S., Riesenhuber, M., & Poggio, T. (2007). Robust object recognition with cortex-like mechanisms. *IEEE Transactions on Pattern Analysis and Machine Intelligence*, *29*(3), 411–426.

Smith, S. M., & Nichols, T. E. (2009). Threshold-free cluster enhancement: addressing problems of smoothing, threshold dependence and localisation in cluster inference. *NeuroImage*, *44*(1), 83–98.

Stelzer, J., Chen, Y., & Turner, R. (2013). Statistical inference and multiple testing correction in classification-based multi-voxel pattern analysis (MVPA): Random permutations and cluster size control. *NeuroImage*, *65*(C), 69–82.

Wang, L., Mruczek, R. E. B., Arcaro, M. J., & Kastner, S. (2015). Probabilistic Maps of Visual Topography in Human Cortex. *Cerebral Cortex* , *25*(10), 3911–3931.
